# Supplementary material for: Kinematics Governing Mechanotransduction in the Sensory Hair of the Venus flytrap
Source: Int J Mol Sci. 2020 Dec 30;22(1):280. doi: 10.3390/ijms22010280 (PMC7795956; doi:10.3390/ijms22010280)
Supplement: Supplementary file 1 [file ijms-22-00280-s001.pdf]

Supplementary material for the article titled - Kinematics Governing Mechanotransduction in the Sensory Hair of the *Venus flytrap*

Eashan Saikia, Nino F. Läubli, Jan T. Burri, Markus Rüggeberg, Christian M. Schlepütz, Hannes Vogler, Ingo Burgert, Hans J. Herrmann, Bradley J. Nelson, Ueli Grossniklaus and Falk K. Wittel

January 6, 2021

1 Supplementary Data S1

The X-Ray CT images of the cross-sections of three Venus flytrap sensory hairs can be found in the dataset bearing the doi 10.3929/ethz-b-000448954 and can be accessed using the following link:
`https://doi.org/10.3929/ethz-b-000448954`

The dataset comprises of a zip file titled HairScans.zip consisting of 3 sub-folders. Each sub-folder is a zip file which contains the cross-sectional .jpg images for a single sensory hair.
